# Dysautonomia in Autism Spectrum Disorder: Case Reports of a Family with Review of the Literature

**DOI:** 10.1155/2011/129795

**Published:** 2011-05-31

**Authors:** Derrick Lonsdale, Raymond J. Shamberger, Mark E. Obrenovich

**Affiliations:** ^1^Preventive Medicine Group/Private Practice, 24700 Center Ridge Road, Westlake, OH 44145, USA; ^2^Department of Pathology, School of Medicine Case Western Reserve University, Cleveland, OH 44106, USA; ^3^King James Medical Laboratory, Westlake, OH 44145, USA

## Abstract

Case histories of a mother and her two children are reported. The mother was a recovered alcoholic. She and her two children, both of whom had symptoms that are typical of autistic spectrum disorder, had dysautonomia. All had intermittently abnormal erythrocyte transketolase studies indicating abnormal thiamine pyrophosphate homeostasis. Both children had unusual concentrations of urinary arsenic. All had symptomatic improvement with diet restriction and supplementary vitamin therapy but quickly relapsed after ingestion of sugar, milk, or wheat. The stress of a heavy metal burden, superimposed on existing genetic or epigenetic risk factors, may be important in the etiology of autism spectrum disorder when in combination. Dysautonomia has been associated with several diseases, including autism, without a common etiology. It is hypothesized that oxidative stress results in loss of cellular energy and causes retardation of hard wiring of the brain in infancy, affecting limbic system control of the autonomic nervous system.

## 1. Introduction

Attention deficit, hyperactivity, a variety of learning disabilities, and autism are often considered to be separate disease entities; however, they also are thought by many to be genetic or epigenetic variations in brain biochemistry [1]. The two children reported here had symptoms of autism spectrum disorder (ASD). Evidence is accumulating to indicate that this spectrum of disorders, appearing early in childhood, responds to nutrient therapy. It has been emphasized [2] that families and clinicians need access to clinical observations and laboratory evidence to assist them in choice of the multiple Complementary Alternative Medicine (CAM) therapies used, a challenge to integrative medicine since pharmaceuticals fail to benefit the primary symptoms and can have marked adverse effects [3]. Of 112 families surveyed, 74% were using CAM for their child with ASD [4]. The high prevalence of this therapeutic approach is often initiated by parents. Clinicians should be prepared to discuss this treatment early in the assessment process [5, 6]. Dysautonomia, involving reduced parasympathetic and increased sympathetic action, was reported in children with autism [7]. 

## 2. Case Reports

A 41-year-old mother had a lifelong history of poor concentration, fatigue, depression, migraine headaches, premenstrual syndrome, seasonal allergy, and endometriosis. She had been treated for many years with fluoxetine hydrochloride. Ingestion of milk caused rhinorrhea. She was a recovered alcoholic and chronically addicted to sugar, ingestion of which was associated with recurrent vaginal yeast infections. Discontinuation of dietary gluten resulted in disappearance of headaches and depression. Both recurred on resumption of gluten. Abnormal thiamine homeostasis was shown by laboratory study. Family history revealed that her mother, grandmother, and sister all suffered migraine headaches and a number of female relatives were described as “obsessive” There was also a sporadic family history of alcoholism.

The 8-year-old daughter had severe personality problems. The pregnancy had been induced for toxemia. At the age of 6 months severe constipation required repeated enemata with occasional megastools. At age of 3 years ureteral reflux required ureteral transplant. She experienced encopresis, seasonal attacks of febrile lymphadenopathy, lethargy, urinary urgency, and attention deficit after ingestion of sugar. Her tongue showed inflammation of the filiform papillae. The heart rate at rest was 104 bpm with marked sinus arrhythmia. Deep tendon patellar reflexes were absent even after reinforcement. Increased concentrations of arabinose and arsenic were present in urine. Intermittent abnormal thiamine homeostasis occurred. Urinary amino acid analysis revealed an overall deficiency of amino acids with the exception of increased concentrations of taurine and anserine. 

The 5-year-old brother had “food allergies” His mother had experienced threatened abortion at 10 weeks, and toxemia had led to induction at term. He had neonatal jaundice, evening “colic” repeated ear infections in infancy and early childhood. Each of the standard preventive inoculations had been associated with prolonged croup. At the age of 1 year he had 10 to 20 myoclonic seizures a day and diarrhea, both of which ceased with gluten and milk withdrawal. Compulsive touching of objects, lack of interest in toys, destructive temper tantrums, and failure of toilet training persisted. He began the repetitive hand flapping movements often observed in ASD, and his behavioral symptoms increased after a pulmonary infection. Urine revealed high concentrations of arabinose and tartaric acid. 

As he grew his behavior included frequent explosive temper tantrums, insomnia, hand tremor, and encopresis related to sugar ingestion. His appetite was otherwise poor. Auscultation revealed evidence of mitral valve prolapse. Deep tendon patellar reflexes were absent without reinforcement. Arsenic was present in his urine. Thiamine pyrophosphate homeostasis was normal until the age of 12 years when its deficiency was demonstrated.

## 3. Methods

**Figure 1:**
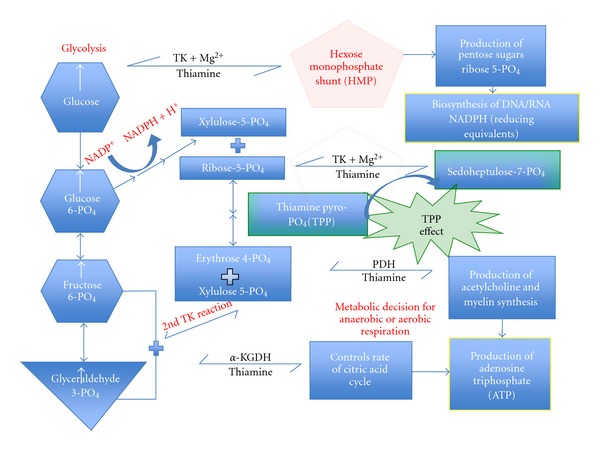
Transketolase requires TPP and magnesium as cofactors. Although the enzyme occurs twice in the hexose-monophosphate shunt, the laboratory test is for the one shown as the TPPE effect. Transketolase TK, pyruvate dehydrogenase (PDH), alpha-ketoglutarate dehydrogenase (*α*-KGDH) Enzymes that all require thiamine (vitamin B1/TTFD) as a cofactor in order to function in carbohydrate metabolism. glyceraldehyde 3-phosphate and fructose-6-phosphate. There are four known natural thiamine phosphate derivatives: thiamine monophosphate (TMP), thiamine diphosphate (TDP) or thiamine pyrophosphate (TPP), thiamine triphosphate (TTP), and the recently discovered adenosine thiamine triphosphate (AThTP). Thiamine pyrophosphate (TPP), also known as thiamine diphosphate (TDP), and cocarboxylase is a coenzyme for several enzymes that catalyze the dehydrogenation (decarboxylation and subsequent conjugation to Coenzyme A) of alpha-keto acids. Thiamine pyrophosphate effect really reflects the saturation status of transketolase with coenzyme.

Erythrocyte transketolase activity (TKA), coupled with the acceleration of its activity by adding thiamine pyrophosphate (TPP) to the reaction, reported as the thiamine pyrophosphate effect (TPPE) is the best way to detect deficiency of TPP [8–11]. Transketolase occurs twice in the hexose monophosphate shunt (HMP) and is dependent on thiamine and magnesium as cofactors (Figure 1). HMP exists in erythrocytes. For the test, reported as TKA and TPPE we used the method published by Massod and associates [12]. The TKA, after controlling for magnesium, measures enzyme activity. The TPPE indicates percentage increase in activity after the addition of TPP. 

 Urinary heavy metal concentrations were measured by direct reading in an Echelle inductively coupled mass spectrometry (Teledyne Leeman Laboratories Inc. Hudson N.H.). Urinary amino acids were measured in the urine of the daughter by liquid chromatography and mass spectrometry (Quest Diagnostics, San Juan Capistrano, CA. Method 36183X,1988) and organic acids by gas chromatography-mass spectrometry (Great Plains Laboratory, Lenexa Kansas).

## 4. Results

Results of repeated TKA, TPPE, and urinary heavy metals are shown in Table 1. Clinical responses in all three subjects, treated with a multitude of vitamin supplements that included thiamin tetrahydrofurfuryl disulfide (TTFD), were variable and unpredictable. Ingestion of sugar, milk, or gluten, that all three individuals craved, produced symptoms immediately. The TPPE in the daughter was unpredictably abnormal, indicating recurrent changes in thiamine pyrophosphate homeostasis. Clinical improvement in her brother occurred early in treatment, mainly by strict restriction of milk, wheat, and sugar. His symptomatic relapses were clearly related to ingestion of any one of these foods. He did not reveal an abnormal TPPE until a followup clinical visit at the age of 12 years when his repeated ingestion of sugar was obviously addictive. The urinary excretions of mercury and arsenic in both children were variable and the source unknown. 

The urine of the boy revealed arabinose (120.3 mmol/mol creatinine: N 0–47) and tartaric acid (155 mmol/mol creatinine: N 0–16). The urine of the girl contained arabinose (294.7 mmol/mol creatinine). An amino acid analysis of her urine showed an increased concentration of taurine (306 mmol/mol creatinine; N 72.2–210.9) and anserine (9.4 mmol/mol creatinine; N zero). All the other amino acids in the sample were substantially below the normal concentration.

## 5. Discussion

### 5.1. Thiamine Homeostasis

Thiamine, normally obtained from diet, has a complex metabolism involving absorption and formation of its three esters, mono-(TMP), di- or pyro-(TPP), and tri-(TTP) phosphates. The action of TPP as a cofactor is well understood but the role of TMP remains obscured. TTP is known to have a fundamental role in the CNS, particularly brain [13–18] but there is presently no clinical laboratory test presently available. TPP has a critical role in the synthesis of cellular energy from glucose. Its deficiency is easily detected by measuring TKA and TPPE [8–11]. Although the laboratory norm for TPPE is given as 0–18%, it may well be that it represents a gradual transition from thiamine sufficiency to deficiency. Because of its occurrence in the hexose monophosphate shunt, decreased TK activity impairs hippocampal neurogenesis, an important part of central autonomic control [19]. Thiamine, being fundamental in energy synthesis, its deficiency produces functional changes, particularly in the CNS. Energy-consuming mechanisms, such as transmethylation and transsulfuration, would be expected to experience loss of efficiency with pathophysiological consequences. What we have shown in the three family members here is that all of them had unpredictable abnormal TPP homeostasis. Clinical observations clearly indicated that there was a profound change in behavior when each of them consumed sugar and it is known that its ingestion and metabolism of glucose increases thiamine requirements [11]. Gibson and Blass have emphasized the role of abnormal thiamine homeostasis in neurodegeneration and have suggested the use of “more absorbable forms of thiamine in therapy” [20]. All three subjects craved sugar, and carbohydrate craving alcohol dependency represents a subgroup of the alcohol-dependent population with distinct personality disorders [21]. Brain white matter growth is highly sensitive to thiamine deficiency [22]. 

### 5.2. Dysautonomia

This term refers to abnormal reflex adaptive reactions of the control mechanisms in the brain and/or in the peripheral distribution of the sympathetic and parasympathetic systems. Dysautonomia has been reported in children with autism [7]. The prototype for nutritionally acquired dysautonomia is beriberi in its early stages of development, now recognized as a form of carbohydrate malnutrition with thiamine deficiency. Degeneration of autonomic ganglia and nerves occurs later in the disease [23]. 

The three family members reported here all had variable symptoms of dysautonomia. Chronic depression and premenstrual syndrome affected the mother, both linked to abnormal autonomic nervous system activity [24, 25]. She had endometriosis, and pelvic pain has been hypothesized to relate to abnormal limbic system activity [26], as are migraine headaches [27, 28]. Pregnancy toxemia has been linked to thiamine deficiency [29]. She had seasonal allergy symptoms, and upper airway inflammatory disease has been related to autonomic nervous system pathology [30]. All three family members had severe symptoms from ingestion of wheat, and the neurological manifestations of celiac disease have been reviewed [31].

The daughter had the kind of constipation with megarectum reported in autism [3] and a history of ureteric reflux requiring ureteric transplant, suggesting that control of neurotransmission was the fault in common. She had repeated episodes of febrile lymphadenopathy, an unusual manifestation of abnormal thiamine metabolism. One of two children reported with these repeated episodes had TPP deficiency [32]. The other had evidence of abnormal TTP metabolism [33]. Supplementary thiamine resulted in cessation of these episodes in both children. The boy in our case report had neonatal hyperbilirubinemia, indicating an almost fourfold risk for infantile autism and oxidative stress [34, 35]. He had prolonged colic, an early expression of some of the most common disorders in childhood [36], including hyperactivity and academic difficulties [37]. He suffered repeated ear infections, a common history in ASD [38], and each inoculation that he received was associated with prolonged croup. Recurrent croup is sometimes associated with gastroesophageal reflux (GERD) [39], a common problem in autism [40]. He started the characteristic hand flapping movements, often observed in ASD children, after he had pneumonia. LoehrI [30] reported that autonomic system dysfunction occurred in patients with chronic upper airway inflammatory disease. A child with recurrent croup had an abnormal TPPE. It ceased with the administration of thiamine (Lonsdale D. Unpublished observation). We hypothesized deficiency of thiamine triphosphate (TTP) affecting the recurrent laryngeal nerve as demonstrated in the phrenic nerve of a victim of Sudden Infant Death Syndrome (SIDS) [41]. Although abnormal TPP homeostasis was not initially demonstrated in K.V., the TPPE was abnormal at the age of 12 years when his ingestion of sugar clearly affected his behavior and loss of wellbeing. Although TTP is important in brain metabolism [42–44], its action is still obscure and there is no readily available laboratory test for its deficiency. 

An additional and unpredictable phenomenon that causes metabolic stress is infection. Recurrent infection, inoculation, and mild head injury triggered episodes of cerebellar ataxia in a child with thiamine-dependent pyruvic dehydrogenase deficiency [45, 46]. Infection triggered intermittent Maple Syrup Urine Disease [47], an inborn error of metabolism that can also be thiamine dependent [48]. Because these intermittent inborn errors of metabolism are rare, their rarity might blind us to considering mild stress of this nature as a trigger in an already unstable state of metabolism in the now common presentation of ASD where there is an unknown genetic or epigenetic risk. Parents of children with ASD are often under the impression that their child's symptoms started after something as apparently benign as a vaccination. It suggests that vaccinations may result in loss of biochemical homeostasis, acting as stressors where there is already genetically determined risk. The boy in the family reported here began his hand flapping movements after he was reported to have pneumonia. He also had prolonged episodes of croup in association with vaccine inoculation.

### 5.3. Energy Metabolism

It has been hypothesized that autism is due to mitochondrial dysfunction [49], supported more recently [50]. Abnormal thiamine homeostasis has been reported in a number of neurological diseases and is thought to be part of their etiology [51]. Blaylock [52] has pointed out that glutamate and aspartate excitotoxicity is more relevant when there is neuron energy failure. Brain damage from this source might be expected in the very young child and the elderly when there is abnormal thiamine homeostasis. In thiamine-deficient neuroblastoma cells, oxygen consumption decreases, mitochondria are uncoupled, and glutamate, formed from glutamine, is no longer oxidized and accumulates [53]. Glutamate and aspartate are required for normal metabolism, so an excess or deficiency are both abnormal. Plaitakis and associates [54] studied the high-affinity uptake systems of aspartate/glutamate and taurine in synaptosomal preparations isolated from brains of thiamine-deficient rats. They concluded that thiamine deficiency could impair cerebellar function by inducing an imbalance in its neurotransmitter systems.

Amino acid analysis of urine from the daughter was performed by parental request. It revealed an increased concentration of taurine and anserine, both involving sulfur metabolism. Deth and associates reported oxidative stress and impaired methylation in autism [55]. Carbon tetrachloride caused a dose-dependent increase in urinary taurine in rats, suggesting that taurine is produced by the liver in response to a toxic insult and subsequent leakage from damaged cells [56]. Taurinuria is dependent on renal sodium chloride transporter activity [57], a mechanism that may be dependent on the action of thiamine triphosphate [58]. Although anserine might be a reflection of meat ingestion, it may be another indication of abnormal sulfur metabolism since it has been shown that carnosine and the related endogenous histidine peptides prevent protein modification such as oxidation and glycation [59].

Arsenic was found in moderate concentrations in the urine of both children and traces of mercury in all three individuals (Table 1). Since these are SH-reactive metals they may have contributed to etiology as additional stress factors. Taurinuria, indicating loss of this amino acid, may have been important in the daughter since rat studies showed that taurine plays a beneficial role against arsenic-induced cerebral oxidative stress [60]. Arsenic exposure induces genomic hypermethylation [61], suggesting the possibility of epigenetic risk.

 Both children had arabinose and/or tartaric acid in their urine. Shaw and associates reported increased urinary excretion of Krebs cycle metabolites analogs and arabinose in two autistic brothers. They showed evidence that this revealed yeast and/or microbial infection responding to treatment with Nystatin [62, 63]. The mother had repeated vaginal yeast infections that she recognized to be associated with sugar ingestion. Although opportunistic fungal pathogens are responsible for life-threatening infections in immunocompromised patients, they are able to persist in immunocompetent individuals through different strategies [64]. A potential source of pathologic behavior from yeast presence has been referred to as the “auto-brewery syndrome” [65], also described more recently in a child with short bowel syndrome [66]. Gastrointestinal disease in autism is common [67]. It has been suggested that there is a possible link between gastrointestinal and behavioral symptoms mediated by innate immune abnormalities [68].

## 6. Conclusion

We have shown several components for pathogenesis in this family constellation. The clinical effect common to all three individuals was abnormal TPP homeostasis and dysautonomia. Although inherited mechanisms are generally considered to be the etiology for dysautonomia [69], it has been hypothesized that oxidative stress, affecting functional efficiency of the autonomic nervous system, is often related to different aspects of nutritional deficiency that damage redox mechanisms [70]. Epigenetics, related to DNA methylation, may also be an important consideration in etiology. 

We hypothesize that it was the contribution of several factors that resulted in clinical expression of disease in these family members. Genetic risk, possibly alcoholism, coupled with the presence of SH-reactive metals in low concentration acting as biochemical stressors and the addictive mechanisms related to the effects of wheat and sugar ingestion had to be in combination. This has been represented as the three circles of health [70]. In spite of its largely unknown action TTP deficiency may play an important part since it is synthesized in mitochondria [71], supporting the conclusion that thiamine is an important nutrient where there is mitochondrial disruption.

## Figures and Tables

**Table 1:** Changes in transketolase activity (TKA), thiamine pyrophosphate effect (TPPE), and urinary heavy metal for the three family members. Normal TKA 42–86 mU/L/min. Acceptable range for TPPE 0–18%. Acceptable range for urinary arsenic 0–45 mg/G. Cr, for mercury, only traces.

	Year	TKA	TPPE	Urine As	Urine Hg
Mother	2004-Dec	31	74%%	23.9	3
	2006-Jul	48	4%		
Daughter	2001-Dec	74	34%	133	4.5
	2002-Jun	62	2%		
	2002-Aug	54	22%	39	0.2
	2002-Nov	82	0%		
	2004-Sept	72	17%	8.8	1.3
Brother	2001-Dec	57	9%	109	2.3
	2002-Feb			222	<0.5
	2002-Jul			26	1.1
	2002-Nov			30	Tr
	2003-Jun			60.4	0.2
	2003-Aug	43	5%		
	2006-Jul	64	6%		
	2008-Oct	42	21%		
